# Investigation of open educational resources adoption in higher education using Rogers’ diffusion of innovation theory

**DOI:** 10.1016/j.heliyon.2022.e09885

**Published:** 2022-07-11

**Authors:** Leila Jamel Menzli, Lassaad K. Smirani, Jihane A. Boulahia, Myriam Hadjouni

**Affiliations:** a Department of Information Systems, College of Computer and Information Science, Princess Nourah bint Abdulrahman University, P.O. Box 84428, Riyadh 11671, Saudi Arabia; b Elearning Deanship and Distance Education, Umm Al-Qura University, Saudi Arabia; c College of Computer and Information Systems, Umm Al-Qura University, Saudi Arabia; d InnoV’COM Lab, University of Carthage, Tunisia; e Department of Computer Sciences, College of Computer and Information Science, Princess Nourah bint Abdulrahman University, P.O. Box 84428, Riyadh, 11671, Saudi Arabia

**Keywords:** Open educational resources, OER Adoption, Higher education, Rogers' diffusion of innovation (DOI) theory

## Abstract

Open Educational Resources (OER) are teaching, and research resources provided under the Creative Commons (CC) licenses and can be freely used, shared, and modified. However, OER adoption is not widespread, and various barriers remain in the way of its growing emphasis. This article is aimed to investigate OER adoption in higher institutions by using Rogers’ Diffusion of Innovation (DOI) theory. 422 responses to an online survey from faculty are gathered and analyzed, where adaptive attributes of DOI are adopted. The results of the descriptive method confirmed that relative advantage has a positive impact on faculty OER adoption. Indeed, positive impacts of observability and complexity are also shown. Ultimately, the findings from the structural model used, indicated that there is a positive correlation between trialability and respectively complexity and compatibility. Whereas relative advantage of OER impacts positively complexity and negatively compatibility. This study showed that it is not enough that faculty agree on OER benefits for teaching and research, the OER adoption rate must increase. Decision-makers in higher institutions are asked to perform additional OER initiatives to overcome challenges related to OER trialability, complexity, and compatibility.

## 1 Introduction

The interest in open access and free use of resources is growing in all domains since there is a global movement toward increasing the dissemination of science and knowledge and sharing it with others without limits or conditions (De Hart et al., 2015). The same trend is felt in the educational field, where interest in Open Educational Resources (OER); including free textbooks, instructional materials, audios, videos, computer applications, and a variety of other tools or technologies, is growing according to UNESCO (Learning, 2011), (De Hart et al., 2015) (Wiley, 2014).

The term OER refers to educational objects, learning or training resources, or research works (De Hart et al., 2015), available as a common public domain or under Creative Common (CC) licenses. These licenses allow the distribution and modification of OER, and promote cooperation with others for reuse, even for commercial purposes (Kim, 2008).

Research in this field is constantly evolving with a special focus on OER challenges about awareness, creation, use. More attention has been given to OER adoption when higher institutions switched to online learning and education due to lockdown regulations imposed during the Covid-19 pandemic period. The adoption rules of OER are generally stated by the nonprofit Creative Commons organization which aims to widen the scope of creative work available for people to exploit and build upon following intellectual property laws (Schloman, 2003). Six licenses protect the work owner (i.e., the way they are dealt with, the methods of distribution, and re-publishing, etc.): CC BY, CC BY SA, CC BY-ND, CC BY NC, CC BY NC SA, and CC BY NC ND, and can be created by many websites (Kim, 2008)] (Schloman, 2003), (Asschenfeldt, 2004) (Venkatesh and Bala, 2008) (Walz, 2018) (Cox, 2018), and (Bourcier and De Rosnay, 2004).

In the educational domain, even though the concept of OER has been introduced and formalized for over two decades, OERs are not exploited in all their benefits and their adoption is still facing many challenges. OER stakeholders are still hesitating on how to use and share open resources due to several factors. Besides the governmental initiatives for OER adoption in higher institutions, faculty members’ engagement on this side remains weak (Sainath Dandawate and Dhanmjaya, 2021).

In the education domain, an innovation is defined as a new or significantly improved product, process, organizational method, or an organization itself developed by or having a significant impact on the activities of a Higher Education institution and/or other Higher Education stakeholders” (Brennan et al., 2014). In alignment with this later, we consider OER use and adoption as an innovation in the higher education context.

The most challenging for every innovation are to investigate the factors influencing barriers and enablers, then the strategies and alternatives to address these factors. This is the case for OER. For this reason, this work aimed to understand the academicians' perceived attributes that impact the adoption rate of all types and formats of open resources.

This study employed the Diffusion of Innovation theory (DOI) of (Rogers, 2003), a theory that concentrates on determinant attributes of users' perceptions and use of innovations. This theory is widely used in many disciplines and considers that actors have the central role in an innovation system that causes the different outcomes of innovation.

This paper is organized as follows: in section 2 we present a review of the literature related to this study. Section 3 describes the research model and hypothesis. Section 4 details the methodology. In section 5 we present the findings based on data analysis. Discussion is conducted in section 6. We end this work with a conclusion and some recommendations.

## 2 Literature review

OER has acquired substantial global traction in recent years as a way of promoting free and open access to academic content. Nevertheless, most scientific studies on OER adoption in higher education are undertaken in the United States, whereas we have not discovered consistent research studies in Asia, or other regions.

### 2.1 OER worldwide initiatives

OER are teaching, learning, and research resources in any medium that are in the public domain or have been distributed under an open license that allows anyone to access, use, adapt, and redistribute them for free with no or few limitations (Learning, 2011) (Tillinghast, 2021). Faculty members' engagement to participate in initiatives is one of the most significant aspects for the success of future education policies. As a result, during the previous two decades, several research projects have attempted to investigate faculty members' levels of engagement in relation to OER adoption and their perceptions of this technology.

The Open Education Group provides a survey of empirical studies on the benefits of OER adoption (Christoforidou and Georgiadou, 2022) (Jones and Nyland, 2020). The Research on OER for Development (ROER4D) project describes OER adoption research across several countries (South America, Sub-Saharan Africa, and Southeast Asia) (The Research on OER for Development, 2022).

Using OER has become an urgent need for worldwide higher institutions as open and shared materials are devastating the learning, research, and training fields. Many initiatives have emerged in the development and dissemination of OER. According to the UNESCO classification (UNESCO, 2019) there are four initiatives levels:

- National Projects: these initiatives are based on the creation and dissemination of OER for the benefit of all educational institutions.
- Community development projects are initiatives launched by a group of people who share a common interest.
- Individual initiatives: these initiatives are taken by individuals who believe in the right to education for all. The most visible of these initiatives is the promotion of Khan Academy's YouTube which aims to offer various teaching materials for everyone and everywhere (Van Allen and Katz, 2020).
- Institutional initiatives: these initiatives are intended for an academic or charitable institution to adopt the development and dissemination of OER.

Despite higher institutions’ initiatives; especially from ranked universities; and ICT capabilities, only a few academics and students adopt OER in teaching and research (Lima-Lopes and Biazi, 2022) (Sainath Dandawate and Dhanmjaya, 2021). This situation has encouraged several researchers to investigate the issues related to OER adoption in the higher educational context (Mayweg, 2021) (Kumar, 2021), (El Kharki et al., 2021) ] (Tang, 2021) (Tillinghast, 2021).

### 2.2 OER adoption in higher education: benefits and barriers

In higher education, many OER sources are available starting from a simple picture to a MOOC, through many search engines (e.g., Google Scholar), universities websites that offer OER (e.g., MIT OpenCourseware, the University of Pittsburgh Library (University of Pittsburgh Library System, 2022) and MOOC platforms (Coursera in the USA, Future Learn in the UK, JMOOC in Japan, Edraak in Jordan, Shams in KSA, etc.) (Massive List of MOOC Platforms Around The World in 2022, 2022).

The faculty community is still balancing between a definitive resistance, partial, and full adoption of OER in teaching and research. The research landscape is evolving in this domain showing OER increasing benefits and barriers to OER adoption.

#### 2.2.1 OER benefits

With the rapid IT growth, many challenges are facing higher education in terms of quality, curriculum outcomes, and students’ employability rates (Kotsiou and Shores, 2021). OER are considered as a solution for higher institutions to face these challenges. In fact, according to UNESCO (UNESCO, 2019) “Universal access to high-quality education promotes peace, long-term social and economic development, and intercultural dialogue.”

In the educational context, most recent studies on OER adoption are conducted in the USA. Yet, other interesting studies were carried out on the effectiveness and barriers of OER adoption in other regions (Nusbaum et al., 2020). The research in (Hilton, 2016) is a synthesis of 16 studies examining the impact of OERs on the quality of higher education and the teachers/students' perceptions of OERs. The results indicated that OER does not appear to influence student learning outcomes: students using OERs achieved the same learning outcomes as the ones using paid resources. Moreover, most students or teachers report a perception that the OER were less likely to help students learn while contributing to saving money. In (Riley and Carmack, 2020) the authors found the same results as in (Hilton, 2016). An improvement in nursing informatics college students’ performances is shown since using OERs contents.

The aim of the study in (Koukis and Jimoyiannis, 2019) was to discover teachers' perceptions of collaborative action to build a MOOC (Massive Open Online Courses) as an open educational resource. Three dimensions were used:(i) individual engagement, (ii) peer interaction and mutual support, and (iii) collaborative creation of educational scenarios and artifacts. The findings revealed that teachers are aware to adopt MOOCs as professional development alternatives. More than this, teachers are willing to adopt other OER as pedagogical practices (for subject contents, knowledge, and professional development).

#### 2.2.2 OER challenges

In (Appiah et al., 2020) a questionnaire was sent to students and faculties of six colleges of the Technical University of Kumasi (KsTU) to measure OERs’ adoption. Five axes are concerned by the descriptive method of this study (i.e., demographic information, knowledge of OERs, location of OERs, use of OERs by students and academic staff, and analysis of attitudes and perception). The authors discovered that 83.9% of academic staff and 91.5% of students have no idea about OER. Besides, there was an indication that the most deterring factors from the use of OER are the lack of training for lecturers on how to use the OER in the KsTU, and inconvenience with the use of the OER library. The study in (Bond et al., 2021) reported the same findings: faculty are reluctant about OER adoption in the Mary Couts Burnett Library at Texas Christian University (TCU) because it is time and energy consuming. Besides, other faculty reported their hesitation to copyrights issues and lack of encouraging climate (funds or awards) for the creation and adoption of OER. The authors indicated that training opportunities and awards/grants from the institutions would be helpful to change the minds of faculty who had considerations or/and difficulties in this subject.

(Kotsiou and Shores, 2021) considered that the significant OER adoption barrier is financial. They recommended three sources for OER funding: private, governmental, and institutional. Moreover, they shared the same ideas as in (Appiah et al., 2020)]. They indicated that (i) faculty are often unaware of what exactly OER and what benefits are they offering for themselves, their students, and their institutions, and (ii) another barrier for OER adoption is the varying and low awareness level of copyrights and Creative Commons licenses from faculty and students. They also share the idea that insufficient training and support is an important issue for fruitful faculty commitment in the OER topic. In (Schuwer and Janssen, 2018)], the authors shared the same upper-mentioned barriers of OER adoption. However, they reported an important cultural issue with sharing learning materials compared with sharing research results, and no immediate negative consequences are seen when using copyright-protected resources. More initiatives are then requested from the Dutch Higher Education Institutions to overcome these barriers.

The authors in (Lantrip and Ray, 2021) aimed to investigate what drives faculty members to utilize OER and what limitations exist in expanding OER options for Oregon colleges. They found that faculty need ongoing support mechanisms for OER adoption, including training on pedagogical best practices and OER technology. They also observed that faculty believed that their efforts had positive impacts for students, like more involvement, student savings, and increased access to education materials. In (Akter and Mahbub, 2020) the authors studied the limitations upon adopting OER from Bangladeshi scholars. They reported that about 50% of academics and scholars agreed that they have a negative attitude in opposition to OER and 50% did not seem to be optimistic about adopting OERs provided by their universities.

The study presented in (Colvard et al., 2018) provided an empirical foundation on which to begin to change the advocacy narrative supporting OER. The authors mentioned that adopting OER has colossal benefits for students: OER improves course grades, reduces withdrawal rates, and consequently increases success rates, especially for part-time students. Moreover, OER leads to significant financial benefits for students and institutions by reducing student debt for expensive textbooks, software licenses and tuitions. Nevertheless, the authors stated that additional research is needed to encourage institutions in higher education in broadening the perception of OER value and leveraging their adoption.

In (Hylén, 2021) the author identified three challenges facing the OER adoption growth:(i) lack of academic copyrights/licenses awareness, (ii) quality of open contents, and (iii) sustainability of institutional OER initiatives for the long run. For (i) the author noticed that besides the several open content licenses developed by the Creative Commons and the GNU Free Documentation License, academics are still hesitant about how to share their work while retaining some rights. For (ii) students and teachers find it difficult to judge the quality and the relevance of open content which hugely impacts OER adoption. For the educational context, institutions have the responsibility to ensure the quality of shared resources to convince users about adopting OER. The sustainability of OER initiatives is essentially due to the growing competition among institutions for funding. Community and institutional models are considered as approaches for this challenge.

A recent systematic literature review of OER in Africa shows that besides being embraced by many institutions, the adoption level of OER is still varying in the region (Tlili et al., 2022). The study findings indicate that most initiatives focused on creating OER, but less effort has been spent on OER adoption in Africa (Tlili et al., 2022). identified five types of challenges that limit the OER adoption in Africa: policies (lack of policies from African governments and institutions, lack of copyright regulations, lack of OER awareness), infrastructure (internet infrastructure and access, lack of educational communities to develop and use OER), financial (lack of awards or citations for the instructors who create their resources as OER, difficulties in ensuring copyright in the distribution and access of resources), pedagogical (lack of skills in creating, searching and applying OER in education, lack of OER licenses interpretation) and personal (lack of time and motivation from teachers in creating or adopt OER, culture and previous believes about free resources use in educational). In this regard, the authors recommend a move away from OER creation to adoption in African countries and a further investigation on cultural and policy challenges for this topic.

In this context, the literature analysis attempted to provide appropriate results on OER adoption in general, and we did not register any exhaustive research projects in the Arab nations. Saudi Arabia, with its OER initiatives and motivations, has the potential to be the locomotive of the Gulf countries in terms of OER spread. Given the same environment and culture, we are certain that the findings of our studies in the KSA will be useful in other golfing countries as well. We conduct this investigation to try to fill a significant gap in the literature.

## 3 Theoretical framework

Several acceptance theories are used to study innovation acceptance and diffusion (Kelly, 2014) (Tillinghast, 2021), (Delone and Mclean, 2003) (Tanye, 2016) (Venkatesh and Davis, 2000) (Venkatesh, 2000), and (Venkatesh and Bala, 2008) with a special focus on people's attitudes about technology (i.e. Theory Reasoned Action (TRA) (Al-Suqri and Al-Kharusi, 2015), Theory of Planned Behavior (TPB) (Ajzen, 2011), Technology Acceptance Model (TAM) is the widely used one (Davis, 1989), which explored determinants influencing user acceptance behavior. TAM was extended by several models (TAM2, TAM3), but it still focused on analyzing situations prior to the time innovation adoption occurs and was criticized to be exhausted (Hester, 2011).

### 3.1 Diffusion of innovation Rogers’ theory (DOI)

The DOI model proposed by (Rogers, 2003) is one of the most used models to analyze the process of communicating any innovation through a systems' members. It focused on explaining post-adoption determinants of innovation. It was and still employed in more than a thousand studies mainly that deal with IT innovations at individual and organizational levels in developed and developing countries (Nazari et al., 2013) (Wang and Wang, 2016) (Rhein, 2021) (Okour et al., 2021) (Ramamurthy et al., 2008) (Oyelana et al., 2021), and (Tsai and Chen, 2022)], In the field of IT systems, Rogers' DOI model presents the strengths to study the adoption process and to explain which factors influence an individual's decision to accept or reject innovation (Kouki et al., 2006).

In the context of OER, DOI theory can lay out the factors formulating the adopter's perceptions of open resources in higher education (Essmiller, 2021) (Almohtadi and Aldarabah, 2021). Faculty's behavior and motivation against OER are subjective and embedded within community and institution context (Schuwer and Janssen, 2018). Besides, Roger's innovation theory is widely used for specific OER like electronic textbooks, MOCC, online databases, Facebook contents, etc., and not for all open resource types or formats. For that reason, this work used the DOI theory to overcome this gap in previous studies.

**Figure 1:**
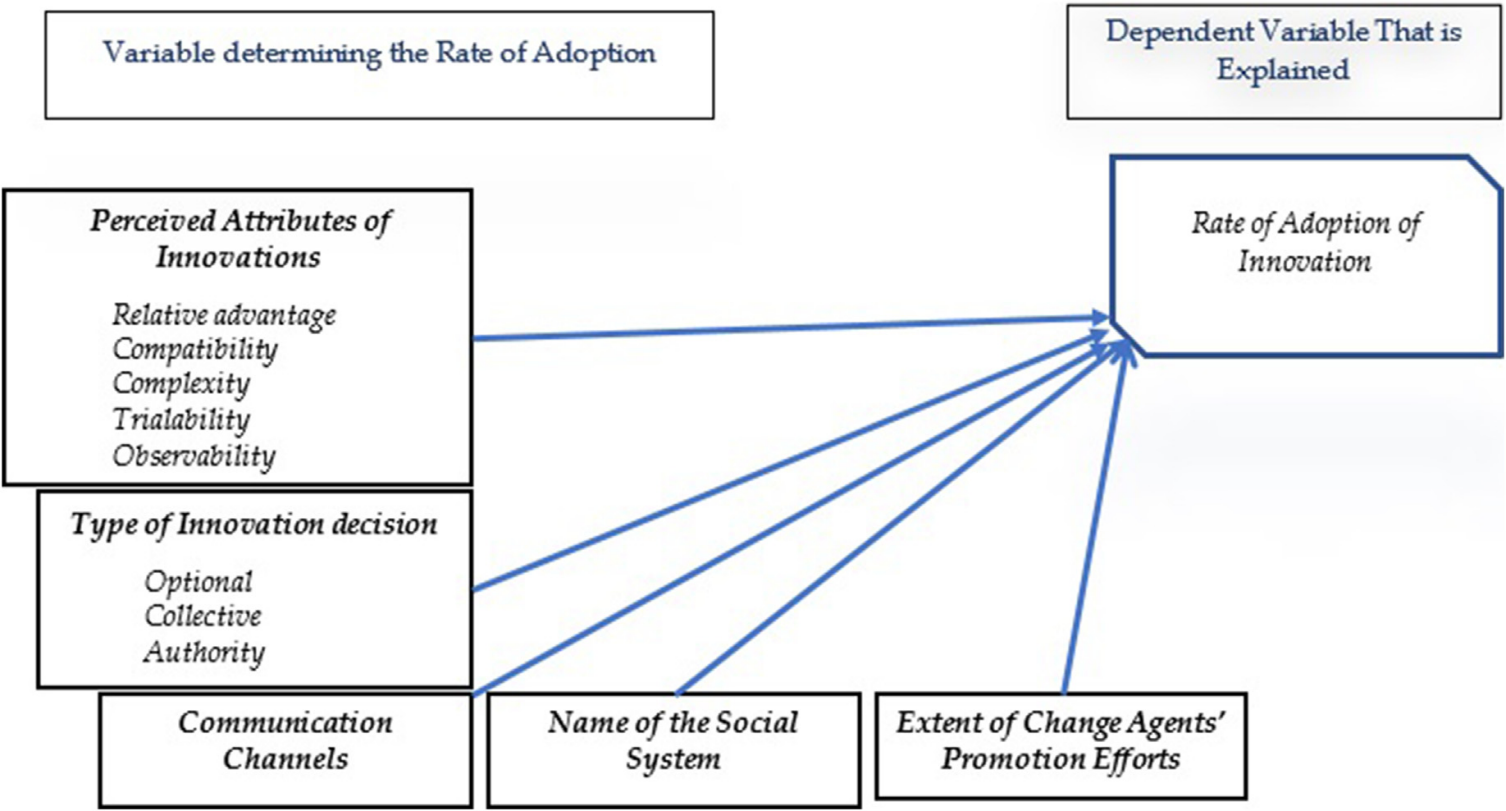
Rogers' Diffusion of Innovation theory Tanye, 2016.

This work is based on the DOI theory which indicated that the diffusion rate of any innovation is highly related to user's perceptions of five important attributes: perceived attributes of innovation, type of innovation-decision, communication channels, nature of the social system, and extent of change agents' promotion efforts (Lundblad, 2003). Figure 1 represents Rogers' DOI.

### 3.2 Research model and hypothesis

**Figure 2:**
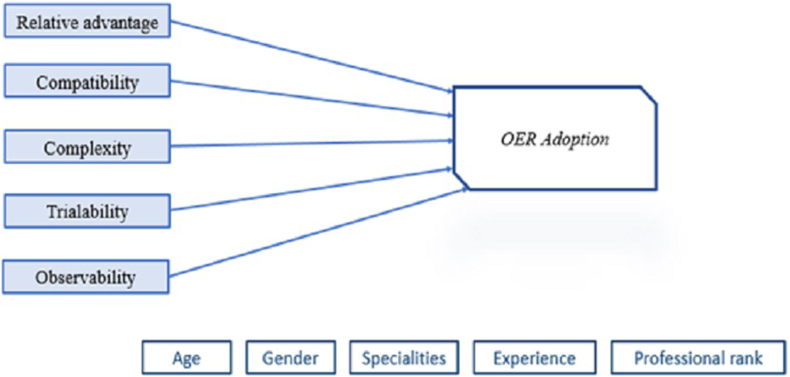
Research model based on the perceived attributes of the DOI.

In this paper, the perceived attributes of the DOI: relative advantage, compatibility, complexity, trialability, and observability are used to investigate faculty's OER adoption (Nazari et al., 2013) as shown in Figure 2.

- Relative advantage is “the degree to which an innovation is perceived as being better than the idea it supersedes” (Rogers, 2003). It is considered as the important determinant of innovations' adoption and reflects how users can benefit from it to perform in their work. In general, for IT systems, the relative advantage is stated to have a positive influence on the IT adoption rate (Al-Ghazali et al., 2015).
- Compatibility is “the degree to which an innovation is perceived as being consistent with the existing values, experience, and needs of potential adopters'' (Tanye, 2016). It integrates the artifacts that suit the work environment of the users with an existing system.
- Complexity is “the degree to which the innovation is perceived as difficult to understand and use” (Rogers, 2003). The innovation complexity is strongly close to the skills and efforts that an adopter needs to find, use, and modify. For faculty, OER finding suitable open resources is time-consuming as the quality of OER contents remains an important issue. Using and modifying OER map with difficulty in understanding Creative Common Licenses and copyrights.
- Trialability is defined as “the degree to which an innovation may be experimented with on a limited basis” (Rogers Everett, 1995). If innovation is widely tried, it is consequentially widely adopted. Regarding OER, most users want to try educational resources in teaching, research, or training before effective adoption to be familiar with Creative Commons licenses.
- Observability is the degree to which the results of the innovation are visible to others (Rogers Everett, 1995). So, the more innovation is visible to other users the more it is adopted. For OER, this can be measured through the results from collaboration in creating, sharing, mixing, and using OER at users' groups' levels with respect to Creative Commons licenses regulations.

This study onward addresses the following hypotheses:

H1 *The relative advantage has a positive impact on OER adoption.*

H2 *The compatibility has a negative impact on OER adoption.*

H3 *The complexity has a negative impact on OER adoption.*

H4 *The trialability has a positive impact on OER adoption.*

H5 *Observability has a positive impact on OER adoption.*

H6 *There is a positive correlation between trialability and complexity.*

H7 *There is a positive correlation between trialability and compatibility.*

H8 *There is a positive correlation between observability and compatibility.*

H9 *There is a negative correlation between relative advantage and compatibility.*

## 4 Methodology

In this study, we use descriptive and analytic approaches to study the phenomena. SPSS26 ensures descriptive research first, followed by structural research using SmartPLS3. A survey was distributed to faculty members of various specialties and different professional ranks, using a Goo questionnaire. The link was distributed to faculties who were conscient that no personal data is collected. Moreover, the respondents were informed about the survey contents and objectives by a brief introductionThe first part of the survey included demographic questions, information about the participants' specialty and years of experience, as well as gender, professional rank, and age. The second part of the survey was made up of DOI attributes (i.e., relative advantage, compatibility, complexity, trialability, and observability). For each attribute a set of related questions is fixed. The structure of the items was based on close surveys made in various studies on OER in higher education. The participators were at the beginning enlightened on the aim of the study, and a definition of each DOI attribute is given.

Based on past research, multiple measuring questions for the questionnaire items were chosen to analyze factors that impact OER adoption. The questionnaire had a total of 28 items; the assertions were graded on a five-point Likert scale, with 1 being "strongly disagree" and 5 being "strongly agree."

Before gathering preliminary data, the survey was reviewed and approved by professionals to ensure that all questions were correctly organized based on the framework's structure. The pretesting was then repeated several times more. The surveys were formerly done once the preliminary tests were approved.

### 4.1 Data collection

The respondents of the survey are faculty members of 25 universities in Saudi Arabia. During this work, no ethical approval is required. Statistical Package for Social Sciences (SPSS) is used to analyze descriptive statistics after receiving 442 valid replies for the academic year 2020/2021.

Table 1 shows the distribution and the demographic features of the sample used. The data was collected by Microsoft from an Excel worksheet for four months (from March to June 2021), then data was coded and analyzed with SPSS. The validity and the reliability of the questionnaire were measured as follows:

**Table 1:** Study's sample demographic features.

Scientific specialization	Frequency	Percent
- Religion sciences	46	10.9
- Administrative, economic and judicial sciences,	273	64.7
- Applied, engineering and computer sciences,	12	2.8
- Human and educational science	43	10.2
- Medical and paramedical sciences,	48	11.4
Professional Rank		
Professor	29	6.9
Associate professor	77	18.2
Assistant professor	231	54.7
Lecturer	76	18.0
Instructor	9	2,1
Gender		
Female	192	45.5
Male	230	54.5
Experience		
Between 2 and 5 years	39	9.2
Between 4 and 10 years	30	7.1
Less than 2 years	103	24.4
More than 10 years	250	59.2
Age		
Between 35 and 55	32	7.6
Less than 35	326	77.3
More than 55	64	15.2
**Total**	**422**	**100**

### 4.2 Questionnaire validity

We use two methods for this measurement, as follows:

- Arbitrators' Competence: the items of the questionnaire in their first version were checked from specialized arbitrators (e.g., axis, clarity, and linguistic phrasing). The language of several of the questionnaire items was changed according to the arbitrators' comments. No item was removed as the agreement on the items of the questionnaire was about 100 percent.
- Internal consistency: the correlation coefficient between the degree of each questionnaire item and the overall score of the axis to which it belongs was determined. The correlation coefficients are statistically significant at the (0.01) level, the correlation coefficient values varied from 0.51 to 0.83, and the internal consistency did not result in the deletion of any items.

### 4.3 Questionnaire reliability

For a sample of 422 faculty members, the reliability was assessed using Cronbach's Alpha method before and after deleting the individual score. Table 2 presents the results obtained.

**Table 2:** Reliability coefficient values of the questionnaire.

Number Of items	Cronbach's Alpha	Cronbach's Alpha if Item Deleted
28	0.851	From 0.818 To 0.851

## 5 Data analysis and findings

The principal goal of this project is to determine what is required to accelerate the adoption of open sharing and reuse of learning resources and open online courses. The desire of faculty, barriers to adoption, and facilitators of acceptance, as well as the impact of institutional and national policy in the adoption of open sharing and reuse of learning materials and online courses, were investigated in this study.

Firstly, to understand differences in motivation to adopt OER, we conducted a series of chi-square tests for discipline, age, gender, professional rank, type of institution, and teaching experience Table 3. The results broadly show that there was a significant difference in motivation between discipline, professional rank, and years of experience, while the other variables had no effect. The Chi-Square Test was used to identify the significance of the differences between the frequencies of the responses of the sample members concerning the items of the adoption of OER, in addition to calculating the arithmetic averages and the agreement percentages of the sample members' responses, and the results were shown in Table 4. The Pearson Chi-square value is less than the significance value (0.05) in all three cases. A Kruskal-Wallis H test showed that there was a statistically significant difference in OER adoption attribute between the different above variables. Consequently, a statistically significant difference i) between faculty's specialty and OER adoption ii) between the professional rank and OER adoption iii) between years of experience and OER adoption.

**Table 3:** Differences in motivation for adopting OER.

	Scientific specialization	Nb	Mean Rank
OER_Adoption	Religion sciences	46	248.05
	Applied, engineering and computer sciences,	273	197.13
	Administrative, economic and judicial sciences,	12	207.75
	Human and educational science	43	279.48
	Medical and paramedical sciences,	48	198.26
	Total	422	

	Professional Rank	Nb	Mean Rank

	Professor	29	152.98
	Associate professor	77	170.68
	assistant professor	231	241.21
	Lecturer	76	189.51
	Instructor	9	172.50
	Total	422	

	Experience	Nb	Mean Rank

	Less than 2 years	39	211.10
	Between 2 and 5 years	30	151.85
	Between 5 and 10 years	103	214.70
	More than 10 years	250	217.40
	Total	422

**Table 4:** Results of Kruskal-Wallis test.

Grouping Variable	OER_Adoption/Scientific specialization	OER Adoption/Years of experience	OER Adoption/professional rank
Kruskal-Wallis H	22.000	7.883	32.607
Df	4	3	4
Asymp. Sig.	.000	.048	,.000

For these three cases, a post-hoc analysis had been conducted to determine which groups differ from each other group. The results are shown in Table 5. The study discovered that there are statistically significant differences for the OER adoption due to scientific specialization, professional rank, and years of experience.

**Table 5:** Differences in the groups extracted by post-hoc analysis.

Grouping Variable	OER_Adoption/Scientific specialization	OER Adoption/Years of experience	OER Adoption/professional rank
Differences in the groups	“Religion sciences” and “Administrative, economic and judicial sciences”. “Religion sciences” and “Medical and paramedical sciences” “Medical and paramedical sciences”, and “Human and educational science” “Applied engineering and computer sciences”, and “Human and educational science”	“Professor” and “Assistant professor” “Professor”, and “Lecturer” “Associate professor”, and “Assistant professor”	“Less than 2 years”, and “Between 2 and 5 years” “Between 2 and 5 years”, and “Between 5 and 10 years” “Between 2 and 5 years”, and “More than 10 years”

Secondly, this study sought to identify the primary reasons behind faculty members' reluctance to use OER**.** The proportions of respondents are nearly identical concerning the ease of use of CC licenses (Table 6). 176 respondents have difficulties using CC, while 118 are unconcerned. This figure is extremely significant, given that the use of OER necessitates some knowledge skills about CC licenses. Thus, the Retention of OER is strongly related to the instructors’ ignorance of OER use rules, especially CC licenses.

**Table 6:** Description of the item related to “It is difficult for me to build a Creative Commons license” (Attribute: Complexity).

	Frequency	Percent	Valid Percent	Cumulative Percent
Strongly Disagree	6	1.4	1.4	1.4
Disagree	89	21.1	21.1	22.5
Neutral	118	28.0	28.0	50.5
Agree	176	41.7	41.7	92.2
Strongly agree	33	7.8	7.8	100.0
Total	422	100.0	100.0	

Third, to identify the role of universities in raising awareness and encouraging faculties to use OER in their academic activities, we investigate faculty members' attitudes toward their universities based on the survey's trialability section. We note that the trend in responses is toward neutrality, with a shrewd agreement on the three items dealing with universities allowing staff to experiment with OER before effective adoption in teaching, research, and training. So, we can conclude that the participants believe that universities don't have an important role in raising awareness and encouraging faculties to use OER in their academic activities.

Fourth, we investigate academic community ideas and behavior about OER. The results in Table 7 show that five clear propositions in the survey haven't been rejected by faculty members: Cpb1 (“I don't accept others to review my educational resources”), Cpb2 (“I don't want to share my resources with others because I spent so much time and effort preparing them”), Cpb3 (“I don't trust other people to mention my name when sharing my resources”), Cpb4 (“I'm afraid of misuse of my own OER”) and Cpb 5 (“When sharing my resources, I'm afraid of misuse of Creative Commons license”). The principal trend in responses is neutral. This is a very important position, and it may be a factor in faculty members' reticence.

**Table 7:** Description of the attribute “Compatibility”.

	Cpb1		Cpb2		Cpb3		Cpb4		Cpb5	
Strongly Disagree	96	22.7%	104	24.6%	43	10.2%	62	14.7%	75	17.8%
Disagree	129	30.6%	118	28%	143	33.9%	81	19.2%	47	11.1%
Neutral	129	30.6%	137	32.5%	115	27.3%	169	40%	157	37.2%
Agree	56	13.3%	39	9.2%	78	18.5%	80	19%	107	25.4%
Strongly agree	12	2.8%	24	5.7%	43	10.2%	30	7.1%	36	8.5%
Total	422	100%	422	100%	422	100%	422	100%	422	100%

Consequently, academic community ideas and behavior about OER are negative factors on OER prediction adoption.

In this part, we calculate the weighted mean and standard deviation of the questions presented in a form like the Likert 5-point scale to know the direction of the respondents' opinions.

Relative Advantage- The general trend of the respondents was to agree and strongly agree on all axes. Table 8 states that the majority of faculty believe that using OER has benefits, such as savings on money, time, and effort. They argue that OER can help them to be more effective with a lower degree of agreement. Also, respondents agree that sharing their resources can improve a university's reputation. The second element, RA2: "You can save effort by using OER" came out on top, then RA1: “You can save money by using OER”.

**Table 8:** The participants' tendency about Roger's attributes.

Roger attributes	Item	Mean	St. dev.
Relative Advantage (RA) (Nazari et al., 2013, Schuwer and Janssen, 2018, Wang and Wang, 2016)	You can save money by using OER	4.18	.717
	You can save effort by using OER	4.19	.752
	You can save time by using OER	4.14	.806
	OER contribute to building students' capabilities	4.03	.767
	Sharing OER enhances my teacher reputation	3.88	.740
	Using OER can enhance the reputation of my university	3.98	.734
	OER help me to be more effective	4.14	.688
Compatibility (Cpb) (Nazari et al., 2013, Schuwer and Janssen, 2018, Wang and Wang, 2016)	I don't accept others to review my educational resources	2.43	1.067
	I don't want to share my resources with others because I spent so much time and effort preparing them	2.43	1.126
	I don't trust other people to mention my name when sharing my resources	2.85	1.148
	I'm afraid of misuse of my own OER	2.85	1.110
	When sharing my resources, I'm afraid of misuse of Creative Commons license	2.96	1.191
Complexity (Cpx) (Nazari et al., 2013, Schuwer and Janssen, 2018, Wang and Wang, 2016)	It is easy for me to use OER	3.86	.867
	I find that dealing with OER is clear and understandable	3.96	.837
	Creative Commons licenses are difficult to find in educational resources	3.35	.886
	It is difficult for me to build Creative Commons licenses	3.33	.942
Trialability (Tri) (Nazari et al., 2013, Schuwer and Janssen, 2018)	I want to try to use OER before effective adoption in teaching	3.62	.895
	I want to try to use OER before effective adoption in research	3.7	.996
	I want to try to use OER before effective adoption in training	3.64	1.061
	My university allows staff to try using OER before effective adoption in teaching	3.38	.957
	My university allows staff to try using OER before effective adoption in research	3.42	.903
	My university allows staff to try using OER before effective adoption in training	3.57	.863
Observability (Obs) (Nazari et al., 2013, Schuwer and Janssen, 2018)	OER provide opportunities to share research with others	4.22	.782
	OER provide opportunities for partnership in teaching	4.2	.700
	OER provide opportunities for sharing the teaching tasks	4.09	.725
	OER encourage cooperative learning	4.20	.679
	Creative Commons Licenses provide the possibility to benefit from the experiences of others	4.09	.608
	I don't see any benefit in mixing other people's work	2.41	1.209

Compatibility-regarding the issue of compatibility, the reader can easily observe that instructors are neutral concerning the following questions Cpb3 (“I don't trust other people to mention my name when sharing my resources”), Cpb4 (“I'm afraid of misuse of my own OER”), Cpb5 (“When sharing my resources, I'm afraid of misuse of Creative Commons licenses”). Conversely, participants present a disagreement about questions Cpb1 (“I don't accept others to review my educational resources”) and Cpb2 (“I don't want to share my resources with others because I spent so much time and effort preparing them”).

Complexity**-**participants show their neutrality regarding either finding CC licenses or the difficulty to build them (Cpx3 and Cpx4). On the other hand, there was a slight agreement regarding the ease of use of OER and about dealing with it, so there is a consensus that OER are simple and understandable.

Trialability**-**six questions are used to evaluate participants' position about OER trialability. Among responses, participants' main desire is to try available OER before integrating them effectively in teaching, research, and training tasks. Yet, they express a tendency to neutrality with the slight agreement concerning universities ‘roles in OER trial use opportunities before operative adoption.

Observability-concerning the Observability attribute, we have four items. For the fifth attribute observability, all responses tend to agree on the first five questions. But for the last question Obs6“I don't understand why merging other people's work would be beneficial”, the trend is in the opposite direction, with disagreement. This observation shows that most faculties recognize the value of OER and are willing to commit to their use and adoption.

### 5.1 ِAssessment of the measurements model

Structural equation modeling (SEM) is a sophisticated multivariate approach that is increasingly being used in scientific research to test and assess multivariate causal links. We used the Smart Partial Least Squares (SmartPLS) as a graphical user interface-based program for variance-based SEM that uses the PLS path modeling approach. This method is commonly utilized in the literature (Chin, 1998). According to a two-step analytic approach, firstly we conduct a psychometric assessment of the measurement scales. Secondly, we use SmartPLS to make the evaluation of the structural model (Hair et al., 2021).

Regarding the evaluation of reflective measurement, relative advantage, compatibility complexity, trialability, and observability were the reflectively measured constructs. Composite reliability (values greater than 70 percent) showed high internal consistency of the constructs.

The result of measurements model (Convergent validity) is obtained for the Outer loading, the Average Variance Extracted (AVE) and the Composite Reliability (CR). The Outer loading of the five DOI constructs is between 0.722 and 0.771 for “Relative advantage”, and between 0.711 and 0.899 for “Trialability”, and between 0.711 and 0.771 for “Observability”, and between 0.704 and 0.842 for” Complexity”, and between 0.740 and 0.886 for |” Compatibility”. The AVE for the five constructs is between 0.524 and 0.816, when the CR is between 0.867 and 0.921.

Convergent validity was assessed by the values of the AVE (all values were above 50 percent) and the outer loading values.

The value of Latent variable correlations is 0.710 for “Relative advantage”, 0.758 for “Compatibility”, 0.730 for “Complexity”, 0.754 for” Trialability” and 0.646 for “Observability.

Discriminant validity was found to be satisfactory for all constructs, as inspection of the cross-loadings showed that each item loaded most strongly on its respective construct (Chin, 1998). Also, we checked the validity of the Fornell-Larcker criterion (Fornell and Larcker, 1981), as detailed in Table 9.

**Table 9:**
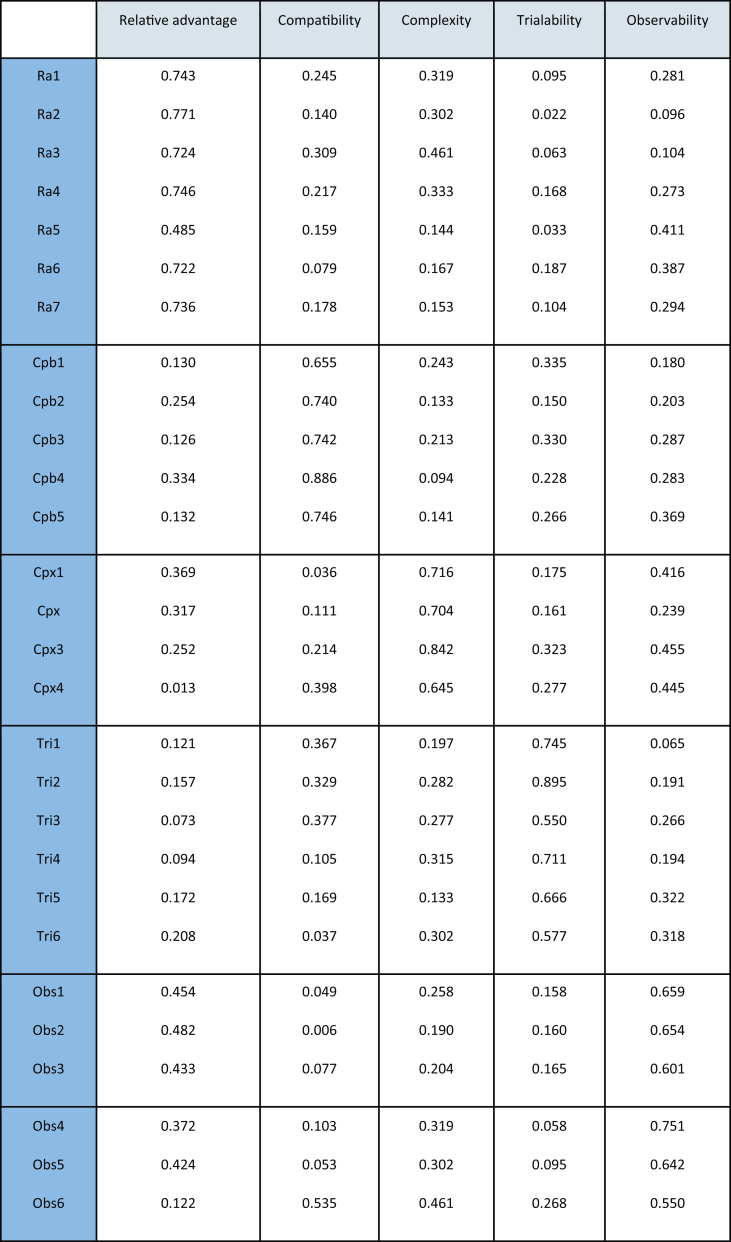
Discriminant validity: Cross loading.

**Table 10:** Path coefficient of the Research Hypotheses.

	Relationship	Std Beta	Std Error	T-value	P-value	Decision
H1	Relative advantage has a positive impact on OER adoption	0.570	0.031	18.127	0.000	Supported∗∗
H2	The compatibility has a negative impact on OER adoption	-0.015	0.020	0.760	0.047	Supported∗
H3	The complexity has a negative impact on OER adoption	-0.218	0.219	7.231	0.000	Supported∗∗
H4	The trialability has a positive impact on OER adoption	-0.004	0.002	0.201	0.841	Rejected
H5	The observability has a positive impact on OER adoption.	0.440	0.431	0.049	0.000	Supported∗∗
H6	There is a positive correlation between trialability and complexity.	0.192	0.404	2.181	0.030	Supported∗
H7	There is a positive correlation between trialability and compatibility.	0.269	0.355	1.951	0.030	Supported∗
H8	There is a positive correlation between observability and compatibility.	0.431	0.408	2.987	0.000	Supported∗∗
H9	There is a negative correlation between relative advantage and compatibility.	-0.439	0.395	2.727	0.000	Supported∗∗

**Figure 3:**
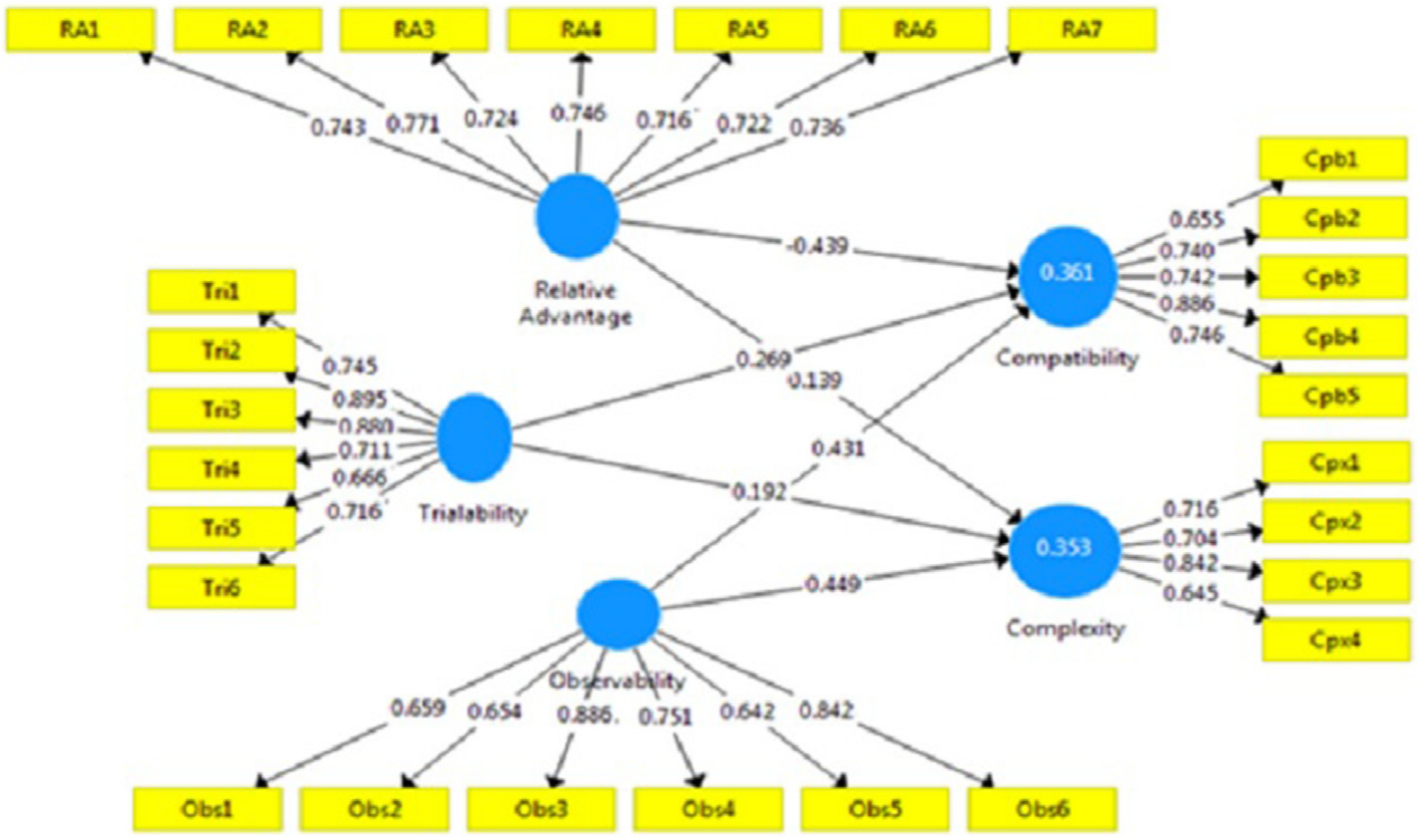
Correlation between DOI perceived attributes.

Figure 3 shows the correlation between the DOI perceived attributes of Rogers’ model.

### 5.2 Assessment of the structural model (Hypotheses investigation)

To investigate the feasibility of our hypotheses, the error probability is specified as the P-value. This means that we can accept the correlation between two attributes if it works 95% of the time. In other words. this relationship may fail 5% (or less) of the time.

Table 10 represents the results obtained with SmartPLS analysis. Except for the fourth hypothesis H4 which is excluded, all the hypotheses H1, H2, H3, H5, H6. H7, H8, and H9 have been retained. Relative advantage and observability with the p-value coefficient of (0.000), and positive Std Beta had the greatest positive influence on OER adoption, whereas complexity with p-value of (0.000) and negative Std Beta of (−0.218) had a negative impact on OER adoption. Compatibility with the p-value of (0.047), and Std Beta of (−0.015) has a negative impact on OER Adoption. Furthermore, the trialability with p-value of (0.841) and Std Beta of (−0.004) had no impact on OER adoption.

The hypothesis H6 is retained as the (p-value and Std Beta) are respectively 0.030 and 0.192). Similar findings for H7 (i.e., p-value equal to 0.030 and Std Beta is equal to 0.269), H8 (i.e., p-value and Std Beta are respectively 0.000 and 0.431) and H9 (i.e., p-value is 0.000 and Std Beta is -0.439).

## 6 Discussion

The primary findings of this work reported no statistically significant differences in OER adoption regardless of age or gender. This argued with (Al-Adwan et al., 2018), (Kim and Lee, 2020) and (Abbad, 2021) works that stated that demographic factors did not influence technology adoption. Yet, we found that there is a statistically significant difference between: faculty specialty and OER adoption; professional rank and OER adoption, and years of experience and OER adoption. These results are significant as to our knowledge, demographic attributes were not considered with the DOI attributes for investigating OER adoption.

The results showed that the DOI attributes have a direct impact on the adoption of OER amongst higher education faculty. This is in conformance with previous studies (Schuwer and Janssen, 2018) (Okour et al., 2021) (Ramamurthy et al., 2008) (Oyelana et al., 2021) (Coleman-Prisco, 2017) (Janardhanam et al., 2011). The results indicated that relative advantage, and observability have significant positive impact, whereas the remaining attributes negatively affect OER adoption rate.

*Relative advantage*-has a direct and significant impact on OER adoption. Similar findings were achieved by (Wang and Wang, 2016) (Rhein, 2021) (Schuwer and Janssen, 2018) (Nazari et al., 2013) (Tanye, 2016), (Almohtadi and Aldarabah, 2021) and (Coleman-Prisco, 2017). In contradiction (Bond et al., 2021), (Colvard et al., 2018) and (Tlili et al., 2022) claimed that relative advantage had no effect on OER adoption, as faculty still believe that creating their own teaching/research materials save more time and effort than finding, modifying, and using OER. An interpretation for these findings, that faculty have different awareness levels (depending on countries, regions, and culture) about the benefits of OER.

*Complexity-*has a negative influence on OER uptake which confirms the hypothesis H3*.* This is in the same line with results reported by most studies. OER adopters would resist as they perceive OER complex for teaching and research. OER needs sufficient skills, time, and effort to understand contents, Creative Commons licenses, and technology (Aggarwal and Makkonen, 2009)].

*Compatibility*-is hypothesized to negatively influence OER adoption. The results of this paper confirm this hypothesis. The compatibility attribute presents a mix of opinions. In (Coleman-Prisco, 2017) faculty believe that OER are compatible with their values and needs of students. Whereas (Martin et al., 2011) and (Brownell and Tanner, 2012) reported that previous ideas and wrong applications of e-learning systems as open resources influence lecturers and students to over adopt or miss-adopt this technology (Martin et al., 2011). Accepting other people's opinions of our educational resources is a mindset of trust in others.

*Observability***-**has a positive impact on OER adoption. This finding is aligned with the (Rogers 2003) and (Greenhalgh et al., 2008) ideas that the more an innovation is visible the swiftly it is adopted. This finding is the same as in (Wang and Wang, 2016) and (Rhein, 2021). This result seems to be logical when new adopters see evidence of use and positive results of OR amongst peers or students. Hester confused technology observability with visibility (Hester, 2011) and reported that it may set out a motivation to adopters to accomplish a feeling of belonging.

*Trialability***-**has no effect on OER adoption for faculty. This means that trialability is not significant for OER adoption rate. This confirms the view of (Hester, 2011), however it contradicts the findings in (Wang and Wang, 2016) (Rhein, 2021), (Schuwer and Janssen, 2018) and (Nazari et al., 2013). This seems to be definitively logical as adopters may or may not require experimenting for a limited time on all types or formats of open resources.

This work confirmed that hypothesis H6, H7, H8 and H9 are supported. For H6 and H7 there is a positive relation between trialability respectively observability and complexity. This seems to be logical as the more experiments of OER by faculty the less the complexity is. Furthermore, once any technology or innovation is highly observed by the community the high the adoption rate. This is in line with the results reported in (Nazari et al., 2013). This could be explained by the fact that faculty are more confident to adopt OER whenever colleagues show positive benefits, results, and feedback from self-experiences.

Hypothesis H8 also, is supported: it is very natural to reach such a conclusion given the concept "observability," which refers to the degree to which OER can be observed by faculty members who are likely to adopt it. If adopters are unaware of OER or do not see it being utilized by their peers, they are less likely to accept it in their existing environment. This has a direct relationship with "Compatibility," which determines how well faculty feel to integrate open resources within existing teaching/research modes and infrastructures. This conclusion confirms the statements of (Hylén, 2021) which reported that OER is stated to raise boosting, prominence, and the pleasure of collaborating with peers, so frustration from faculty against OER technology and environment is reduced.

Hypothesis H9 states that there is a negative correlation between relative advantage and compatibility. The choice to accept OER is a dynamic process that involves interactions between the original authors, those who made modifications, and those who remixed. All of this is controlled by CC licenses, as well as situational and contextual circumstances and resource aspects. Faculty members who participate in technology adoption are not rational participants, and the benefits and drawbacks of OER are dispersed unevenly among those involved. Adopters, on the other hand, contribute their own set of interests, values, and power, all of which influence and complicate the process of adoption. This finding may be the subject of further analysis in future work to improve our understanding of these many elements of impact and to provide useful information to guide OER's extension efforts.

## 7 Conclusion

The main objective of this work was to investigate the attributes that determine the OER adoption by faculty in the higher education field. The results showed that demographic attributes have no significant impact on the rate of open resources adoption. The results indicated that the perceived attributes of Rogers innovation theory are helpful to investigate the adoption of all types and formats of OER amongst faculty in 25 of Saudi universities. This study indicated that all faculty argued on OER benefits as saving time, money, effort, opening to the world, and best curriculum outcomes.

Nevertheless, the participants mentioned that they are still reluctant to fully create, adopt or use OER for research or teaching. This is particularly shown through the weak involvement of the Saudi faculty in the OER Shams platform. This is mainly because of the complexity and the lack of trialability of OER. Indeed, compatibility and observability issues are also barriers to more adoption of OER.

Given these findings, several recommendations may be suggested to encourage decision makers and faculty in higher institutions to overcome the above issues and raise OER adoption rate. At an individual level, faculty must invest in creating, sharing, adopting OER in their courses and research. Moreover, faculty are requested to make more self-efforts to overcome traditional and negative thinking about OER. At the community level, faculty are asked to motivate others in the same institution by sharing more OER contents and benefits of adoption experiences. Finally, we think that the most recommendations are at the institution level. Universities must provide faculty and students with more awareness conferences and training programs to master how to create, use and adopt OER with respect to Creative Common licenses. Higher institutions are requested to intensify the OER platforms. Indeed, faculty need awards and recognition from their institutions to raise involvement in this topic.

To summarize, the issue of adopting OER remains a mental challenge, considering co-authorship necessitates a high level of openness to the academic and educational communities in general. Likewise, even if revision and modification are required, there must be a minimum of conviction in the work of the other. As a result, each OER usage necessitates an investigation into the content and a review of the material, as well as the content adapting to the use.

The sharing of open educational materials, on the other hand, is a process in which others benefit and gain from the contribution of others. Faculties cannot be satisfied only by consuming the materials available on the various open digital platforms; they should also participate and contribute to the creation of content and its distribution to others. Considering the preceding, the study strongly advises institutions to raise faculty members awareness to the OER value and to build new methods for the management of educational lessons implementation, since change necessitates many outreach efforts.

Awareness must help people understand how OER may improve educational outcomes, save time, and reduce costs. Moreover, higher institutions must work hand in hand with national and international OER communities to optimize digital platforms for free licenses so that the OER can be found easily, facilitate translation and adaptation of content to cultural context. We also think that more effort is needed to boost OER construction by faculties and students with deep advice and support on author's rights and creatives commons licenses.

This work presents the limitation of considering a small sample of 422 faculty members from only 25 universities, i.e. about 60% institutions replied to the survey (from 42 government and private universities); and belonging to the same country (i.e., participants share same habits, traditions and attitude regarding OER). We think that responses from large number of faculties with heterogenous backgrounds may give different results. The findings could be further generalized and realistic. Indeed, the investigation has been conducted during the Covid 19 pandemic when all academic activities were in fully online mode. This may have had an influence on responses as academic participants were more resistant or open to OER adoption, or lightly committed to online surveys because the ‘screen tiredness” during this period. Another limitation is that adoption of OER from home institutions are also evaluated in this study. We think that this may impact the responder's objectivity towards OER. The study used a survey to gather faculty responses. We believe that other gathering tools could be used, as interviews with faculty focusing groups; according to OER faculty involvement (i.e., creating, reusing, sharing, etc.) or OER types (videos, courseware, audios, modules, etc.)

Finally, the limitation is the scarcity of similar research in this field which makes the comparison of the results hard. So other research is essential to settle the findings of this study in the same country or in similar regions.

We think that there is still working to investigate OER adoption in higher education. Other acceptance theories may be used, such as the Unified Theory of Acceptance and Use of Technology (UTAUT) to fix additional determinant attributes. Additionally, considering the OER adoption from students' side may be an interesting field to raise worldwide OER initiatives and reach before the beginning of 2030 “open resources is for everyone and everywhere”. Ultimately, we think that other OER adoption barriers are to investigate in the future like content quality issues, adoption process, adopters OER interests, and OER adoption and accessibility for persons with disabilities, etc.

## Declarations

### Author contribution statement

Leila Jamel Menzli; Lassaad K. Smirani: Conceived and designed the experiments; Performed the experiments; Analyzed and interpreted the data; Contributed reagents, materials, analysis tools or data; Wrote the paper.

Jihane A. Boulahia: Analyzed and interpreted the data; Contributed reagents, materials, analysis tools or data; Wrote the paper.

Myriam Hadjouni: Conceived and designed the experiments; Performed the experiments; Analyzed and interpreted the data; Wrote the paper.

### Funding statement

This work was supported by Princess Nourah bint Abdulrahman University Researchers Supporting Project number (PNURSP2022R193), Princess Nourah bint Abdulrahman University, Riyadh, Saudi Arabia.

### Data availability statement

Data will be made available on request.

### Declaration of interests statement

The authors declare no conflict of interest.

### Additional information

No additional information is available for this paper.
